# Modified aluminum (Al)-hematoxylin stain for detection of Al in sheep and cat tissues: an animal model for the study of Al-associated conditions

**DOI:** 10.1007/s11259-025-10679-y

**Published:** 2025-02-19

**Authors:** Estela Pérez, Alicia de Diego, Álex Gómez, Ana Rodríguez-Largo, Marta Pérez, Lluís Luján

**Affiliations:** 1 Departamento de Patología Animal, Universidad de Zaragoza, Zaragoza, Spain; 2 Instituto Universitario de Investigación Mixto Agroalimentario de Aragón (IA2), Universidad de Zaragoza, Zaragoza, 50013 Spain; 3 Instituto Aragonés de Ciencias de la Salud (IACS), Zaragoza, 50009 Spain; 4 Departamento de Anatomía, Embriología y Genética, Universidad de Zaragoza, Zaragoza, 50013 Spain

**Keywords:** Aluminum, Granuloma, Adjuvant, Sheep, Feline Injection Site Sarcoma, Cat, animal model, Histochemistry, Pseudolymphoma

## Abstract

Aluminum (Al) has been an effective adjuvant in vaccines for nearly a century in animals and humans. Al can induce persistent granulomatous inflammatory responses characterized by macrophages with large granular, blue-gray cytoplasm sometimes with intracytoplasmic structures known as crystalloid bodies. These granulomas have been linked to proliferative and neoplastic conditions at injection sites in animal and humans. The detection of Al in tissue is crucial but current methods are time-intensive and require specialized training. This study highlights the efficacy of the modified Al-hematoxylin (MAH) technique in animals. Tissues from Al-induced lesions in sheep and cats were examined, along with non-aluminum granulomas as controls and MAH results were confirmed with lumogallion staining. In sheep, MAH positively stained granuloma macrophages, crystalloid bodies, necrotic regions and nearby connective tissue macrophages, while lymphoid aggregates present in granulomas were consistently negative. Macrophages loaded with Al and crystalloid bodies in lymph nodes were also MAH-positive, as were macrophages in contaminated injection sites following application of commercial Al-based vaccines. In cats with Feline Injection Site Sarcoma (FISS), MAH detected macrophage clusters around the tumor periphery and within the sarcoma. This cost-effective, rapid, and specific technique provides valuable insights for diagnosing Al-related conditions and could serve as an animal model for other species, including humans.

**Supplementary Information:**

The online version contains supplementary material available at 10.1007/s11259-025-10679-y.

## Introduction

Since the serendipitous discovery of the aluminum (Al) effect on the immune system, aluminum-based adjuvants (ABAs) have served as a cornerstone of immunopotentiation in millions of animal and human vaccines for nearly a century (HogenEsch et al. 2018). The efficacy of Al as an adjuvant is well documented, and it remains a critical component of numerous vaccine formulations (HogenEsch et al. 2018; Frings et al. 2021), particularly in veterinary vaccines (Domínguez-Odio et al. 2022). However, vaccination with Al can induce localized pruritic, focal granulomatous inflammatory reactions, which may exhibit necrotic centers in both animals (Hendrick and Dunagan 1991; Valtulini et al. 2005; Day et al. 2007; Asín et al. 2019) and humans (Slater et al. 1982). In these granulomas, ABA particles are consistently found within macrophages with large, granular, blue-gray cytoplasm (Valtulini et al. 2005; Asín et al. 2019), sometimes forming large, rod- or cigar-shaped intracytoplasmic structures known as crystalloid bodies (Asín et al. 2019). This granulomatous reaction may persist locally for months or even years (Valtulini et al. 2005). In routine veterinary pathological diagnosis these granulomas are often identified in surgical biopsies during the evaluations of potential neoplasms (Gross et al. 2005a). Notably, granulomas at injection sites have been associated with proliferative conditions such as soft tissue sarcomas in cats, dogs, birds, and ferrets (Hartmann et al. 2015; Day et al. 2022), as well as pseudolymphomas in dogs, cats and humans which may progress to lymphomas (Gross et al. 2005b). In humans, studies consistently demonstrate an association between Al and pseudolymphomas (Frings et al. 2021), and there are suggestive findings linking vaccines to feline cutaneous pseudolymphomas or lymphomas, though conclusive evidence remains lacking (Roccabianca et al. 2016). The presence of Al in granulomas and/or proliferative lesions is often hypothesized, with publications often relying primarily on the location of injection sites, clinical history, and/or morphological characteristics of the lesions (Couto et al. 2002; Day et al. 2022). Available histochemical techniques such as aluminon and solochrome azurine have occasionally been applied to injection site granulomatous reactions and tumors (Vascellari et al. 2003; Valtulini et al. 2005); however, both methods exhibit potential limitations in specificity and/or sensitivity (Fernández-Martín et al. 1996), and are not commonly performed in most research and diagnostic laboratories. Other available, more specific techniques require specialized training, resources and are time consuming (Asín et al. 2019; Mold et al. 2019; Frings et al. 2021). Among these, lumogallion fluorescence staining has been used as a gold standard for validating other Al visualization methods (Mold et al. 2019). A simple, effective histochemical detection method for Al in tissues would be highly beneficial for both diagnostic and research purposes in veterinary and human medicine (Frings et al. 2021).

In this study, hematoxylin alone was used to specifically detect Al in mammalian tissues, based on previous reports of Al detection in plant roots, freshwater organisms (Havas 1986), and trout organs chronically exposed to Al (Exley 1996). We refer to this histochemical technique as modified Al-hematoxylin (MAH). The aim of this study is to demonstrate the simplicity and specificity of the MAH technique for detecting Al in sheep and cat tissues, providing an animal model that could also be applied to studying Al in other mammalian species, including humans.

## Materials and methods

### Study cases

A summary of the study cases and stains used in this work is provided in Supplementary Table S1. The test cases included Al-containing tissues from lambs experimentally vaccinated against bluetongue virus (BTV; Study cases 1 and 2), as well as Al-containing tissues (including granulomas) from commercial vaccines administered to sheep (Study cases 3 and 4). Additionally, a group of accidentally contaminated injection sites, following routine application of commercial Al-based vaccines was included (Study case 5). Study case 6 involved tissue biopsies from cases of feline injection site sarcoma (FISS) cases. Control cases included tissues from experimental vaccines against BTV that did not contained Al based adjuvants (Study cases 7 and 8), tissues from experimental injections of inactivated BTV alone (Study cases 9 and 10), and tissues from experimental injections of PBS (Study case 11). Additional negative control tissues were obtained from natural cases of granulomatous enteritis induced by *Mycobacterium avium* subsp. *paratuberculosis* infection in sheep (Study case 12), as well as experimentally induced granulomas in rabbits using Freund’s adjuvant (Study case 13). Some of the samples were from experiments previously approved by the Ethical Committee of the University of Zaragoza (ref. PI34-18 and ref. PI30-19). Other test cases were retrieved from the archives of the Department of Animal Pathology, University of Zaragoza, and involved animals submitted for postmortem examination by referring veterinarians. All cases were selected, fixed in 10% neutral buffered formalin, embedded in paraffin, and sectioned into 4 μm-thick slices for staining with hematoxylin-eosin (H/E), MAH, or lumogallion.

### Modified Al-hematoxylin stain (MAH)

The MAH stain was performed following previously published protocols (Havas 1986; Exley 1996). This method employs oxidized, unmordanted hematoxylin, also known as hematein. In contrast to traditional Al-mordanted hematoxylin staining, this method utilizes the Al endogenously present in tissues as the mordant, specifically highlighting Al-binding sites for hematein with a deep purple stain. Briefly, 0.12 g of crystalline hematoxylin (Sigma-Aldrich, Germany, ref. C.I. 75290) and 0.01 g of sodium iodate (Sigma-Aldrich, Germany) were dissolved in 50 ml of distilled water, producing an amber-colored solution. Tissue sections were deparaffinized, hydrated, and immersed in this solution for 20–30 min, followed by dehydration and mounting.

### Fluorescence Al-detection (Lumogallion)

Lumogallion staining was conducted as previously described (Asín et al. 2019). Fluorescence images were visualized and captured using an inverted fluorescence microscope (Olympus IX81) equipped with a U-MNIBA3 filter cube (excitation filter 470–495 nm; beam splitter 505 nm; barrier filter 510–550 nm). For each sample, a consecutive control section was included to ensure consistency.

## Results

**Fig. 1:**
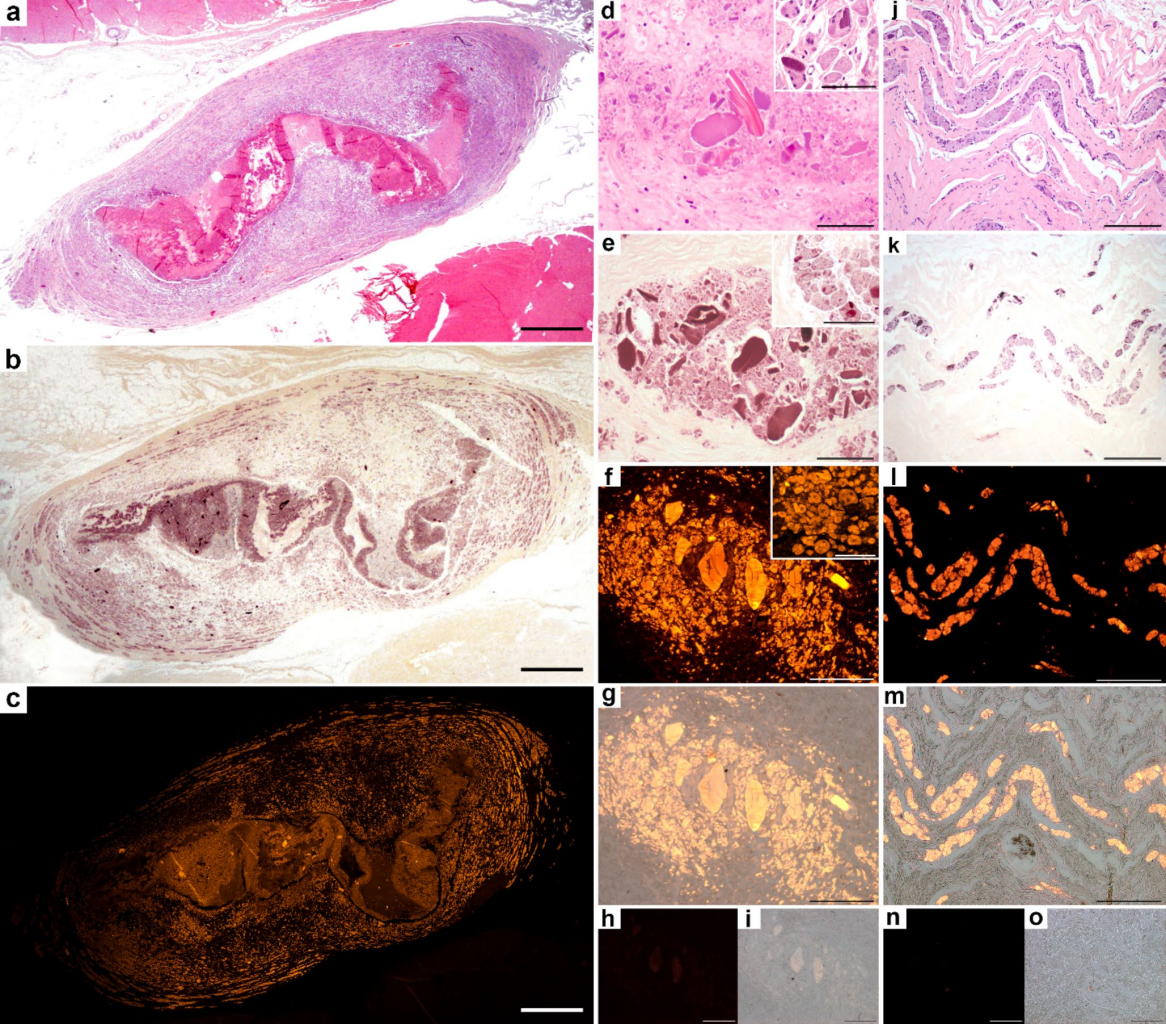
Sequential sections of experimental, aluminum (Al)-induced, subcutaneous post-vaccine-granulomas in sheep. (**a**) Granuloma structure with a central elongated area of necrosis surrounded by layers of macrophages and fibrous tissue. Hematoxylin-eosin (HE). (**b**) Al presence is detected as deep purple staining throughout the granuloma, most intense at at the necrotic center and the surrounding cells. The remaining tissue exhibits a pale-yellow color. Modified Hematoxylin (MAH). (**c**) Al presence is confirmed by fluorescent staining, showing the same distribution as the MAH stain. Yellowish-orange aggregates are identified as Al, while the rest remains black. Lumogallion. **a-c**. Bar: 1 mm. **d-f**. Serial sections of an Al-containing granuloma. (**d**) Formation of Al crystalloid bodies within a granuloma, with large aggregates of Al intermingled with Al-laden macrophages. *Inset*: Macrophages with granular, basophilic cytoplasm, sometimes with crystalloid bodies. HE. (**e**) Al presence is confirmed by a deep purple color in both, large aggregates and macrophages, with the rest of the tissue remaining unstained. *Inset*: Detail of Al-laden macrophages. MAH. (**f**) Al crystalloid bodies and macrophage cytoplasms exhibit a yellowish-orange color that confirms the presence of Al. *Inset*: Detail of Al-laden macrophages. Lumogallion. **j-l**. Serial sections of the granuloma periphery. **j**. Bands of activated macrophages intermingled with connective tissue. HE. **k**. Matched, specific staining of Al within macrophages. MAH. **l**. Al presence is confirmed in these activated macrophages. Lumogallion. **g**,** m**. Lumogallion fluorescence merged with bright-field channel. **h**,** n**. Negative controls (fluorescence channel). **i**,** o**. Negative controls (fluorescence channel) merged with bright-field channel. **d-o**. Bar: 200 μm. **d-f**. *Inset* bar: 100 μm

Granulomas induced by experimental injection of Al-based vaccines in sheep (Study case 1, Suppl. Table S1) exhibited a concentrically multilayered structure, with a central necrotic core, layers of activated macrophages, and multinucleated giant cells displaying granular basophilic cytoplasm. These structures were surrounded by scattered lymphocytic aggregates and a well-defined fibrous capsule encasing the granuloma (Fig. 1a). MAH staining revealed multiple intense deep purple deposits in the cytoplasm of both macrophages and giant cells, as well as within the necrotic areas, against a paled yellow background (Fig. 1b). Lumogallion staining confirmed the distribution observed with MAH and the fluorescence corresponding to Al aligned perfectly with the MAH results in all samples from Study case 1 (5/5; 100%) (Fig. 1c). Additionally, large intracellular crystalloid bodies were present in the cytoplasm of these macrophages (Fig. 1d). Both MAH and lumogallion stains consistently demonstrated the presence of Al in the macrophage cytoplasm and crystalloid bodies (Fig. 1e-g), while control samples exhibited only mild autofluorescence (Fig. 1h-i). Macrophages interspersed in the connective tissue adjacent to the granulomas were readily identifiable by their morphology (Fig. 1j), and consistently stained positive using both MAH and lumogallion techniques (Fig. 1k-o).

**Fig. 2:**
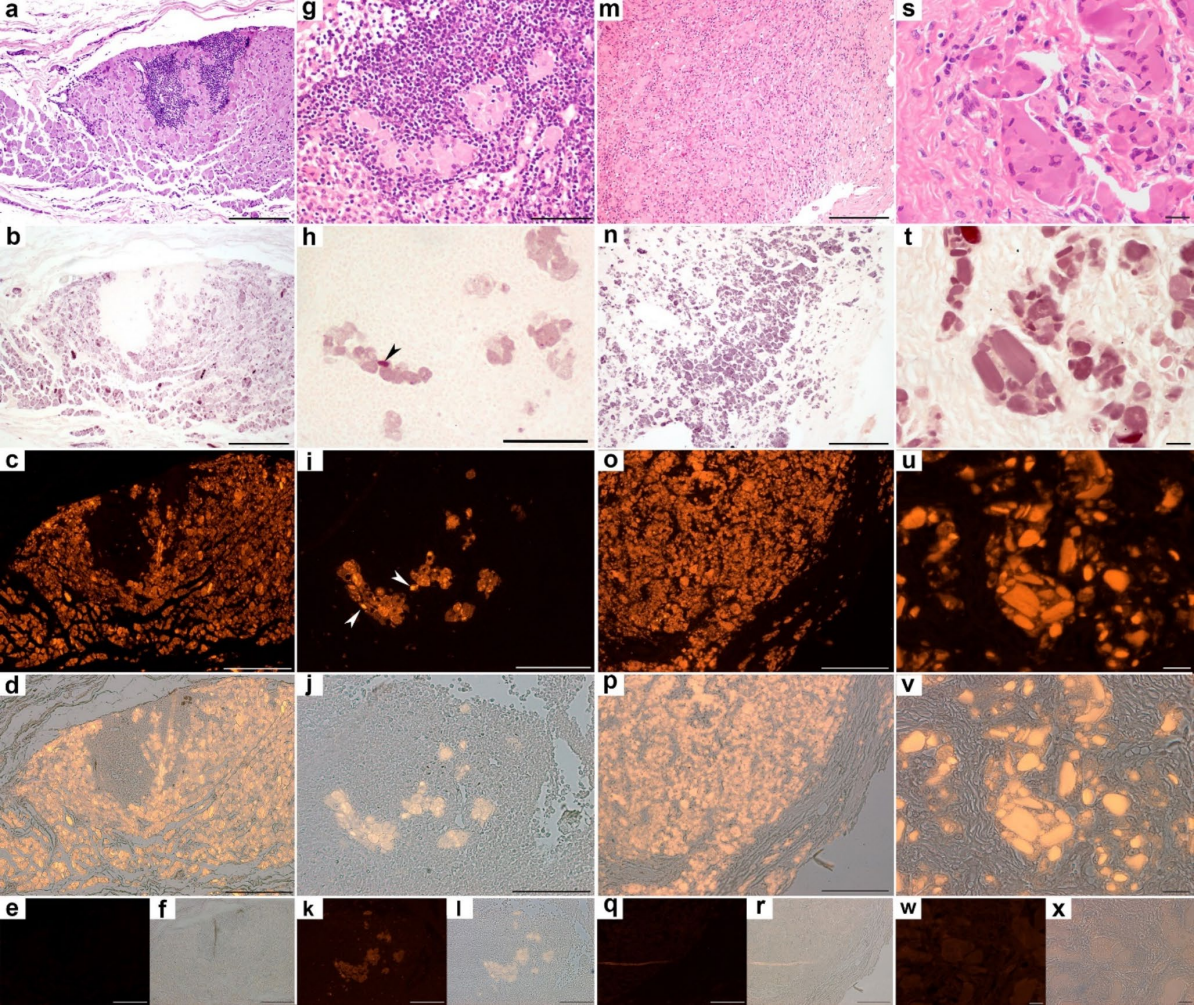
Sequential sections showing the presence of aluminum (Al) in sheep tissue. **a-c**. Al-induced granuloma with a tertiary lymphoid follicle. (**a**) Large lymphoid aggregate within a granuloma. Hematoxylin-eosin (HE). (**b**) Al presence (deep purple color) within activated macrophages surrounds lymphocytes, that are negative for the staining. Modified Al-hematoxylin (MAH). Note the presence of Al-positive macrophages immersed within the lymphocyte aggregate. (**c**) Fluorescence matches and confirms the location of Al and the absence of the metal in lymphocytes. Lumogallion. **g-i**. Activated macrophages within a lymph node. **g**. Large cells corresponding to macrophages are located between lymphocytes. **h**. Specific localization of intracytoplasmic Al in the lymph node; note the crystalloid body (*arrowhead*). MAH. **i.** Matched results obtained with fluorescence; several crystalloid bodies are indicated (*arrowheads*). Lumogallion. **m-o**. Subcutaneous granuloma induced by a commercial vaccine. **m**. Multiple macrophages present in the connective tissue. HE. **n**. Most macrophages are loaded with Al, as demonstrated by a deep purple staining. MAH. **o**. Al presence in macrophages confirmed by fluorescence. Lumogallion. **s-u**. Detection of Al in a case of contamination after vaccination. **s**. Large macrophages with granular cytoplasm and formation of crystalloid bodies. HE. **t**. Presence of Al indicated by a deep purple staining. MAH. **u**. Confirmation of Al within macrophages by fluorescence. Lumogallion. **d**,** j**,**p**,** v**. Lumogallion fluorescence merged with bright-field channel from images c, i,o, u, respectively. **e**,** k**,**q**,** w**. Negative controls (fluorescence channel). **f**,** l**,**r**,** x**. Negative controls (fluorescence channel) merged with bright-field channel. **k**,** l**. Mild autofluorescence is observed. **a-f**. Bar: 200 μm. **g-l**. Bar: 100 μm. **m-r**. Bar: 200 μm. **s-x.** Bar: 20 μm

Four granulomas from Study case 1 also contained embedded tertiary lymphoid aggregates (Fig. 2a), which were negative for both MAH and lumogallion staining (4/4; 100%) (Fig. 2b-c). Embedded within these lymphocyte-rich areas only isolated macrophages exhibited staining, confirming specificity (Fig. 2a-f). In axillary lymph nodes, Al-loaded macrophages were observed as clusters or individual cells containing intracytoplasmic Al aggregates most cases with occasional intracytoplasmic crystalloid bodies (Fig. 2g-i). These Al-laden macrophages stood out against a yellow (MAH) or black (lumogallion) background (Fig. 2h-l), both stains fully coinciding (9/9; 100%). MAH staining successfully detected Al in granulomas induced by commercial vaccines in sheep, as confirmed by lumogallion staining, both stains fully coinciding (3/3; 100%) (Fig. 2m-r). Accidentally contaminated injection sites following the use of commercial Al-based vaccines displayed a range of inflammatory components including fibrin, neutrophils, extensive necrosis, cellular debris, and bacteria. Despite this complexity, MAH successfully detected Al within the cytoplasm of macrophages and giant cells, a finding that was also confirmed by lumogallion in all samples tested (10/10, 100%) (Fig. 2s-x).

**Fig. 3:**
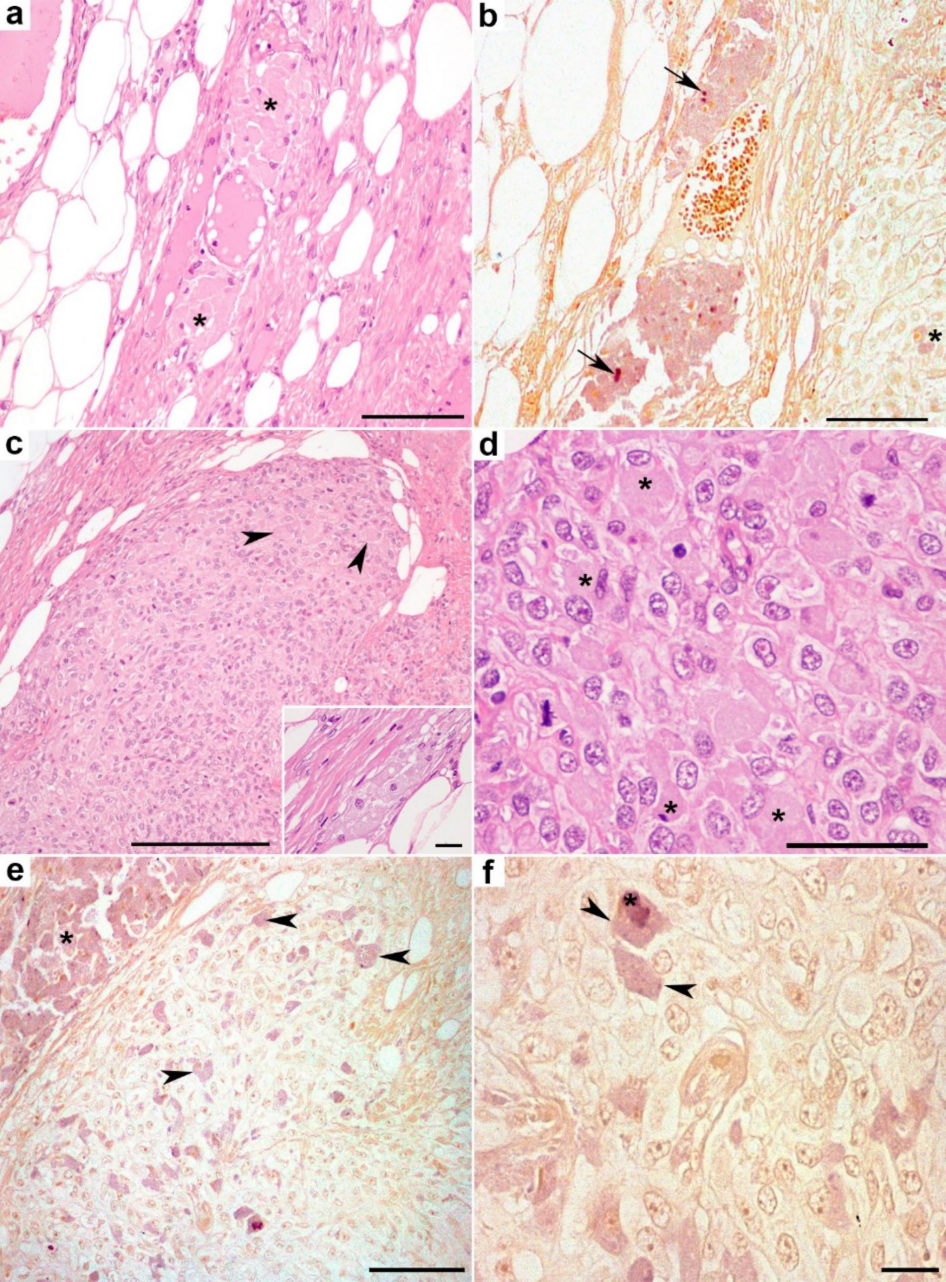
Histopathological images of a cat with Feline Injection Site Sarcoma (FISS). (**a**) Tumor margin: Perivascular aggregates of macrophages showing large granular cytoplasm (*asterisks*). HE. Bar: 100 μm. (**b**) Consecutive section of (a); macrophages exhibit deep purple cytoplasmic staining indicative of aluminum (Al) content. Surrounding tissues show pale, yellow background staining. Small crystalloid bodies (not visible with HE) demonstrate positive, deep purple staining (*arrows*). Modified Al-hematoxylin (MAH), Bar: 100 μm. (**c**) Sarcoma areas located at the periphery of the tumor. Embedded in these areas are cells with histiocytic morphology and large granular cytoplasm (*arrowheads*), resembling those shown in (a). HE. Bar: 100 μm. *Inset*: clusters of macrophages within the capsule. Bar: 20 μm. (**d**) Differentiated, histiocytic-like cells with large, eosinophilic granular cytoplasm (*asterisks*) found within the sarcoma. Surrounding cells show clear cytoplasm. HE. Bar: 50 μm. (**e**) Scattered intratumoral MAH positive cells are distinguished by deep purple staining (*arrowheads*). Note the presence of an aggregate of MAH positive macrophages (*asterisk*). Adjacent sarcoma cells remain negative to the presence of Al. Nuclei and surrounding tissues show yellow staining (negative to MAH). MAH. Bar: 100 μm. (**f**) Histiocytic cells showing deep purple granular cytoplasm (*arrowheads*) and occasional intracytoplasmic crystalloid bodies (*asterisk*). Bar: 20 μm. MAH

Only in one cat (Study case 6, Suppl. Table S1), aggregates of macrophages with granular cytoplasm were observed with HE at the tumor periphery, either near or within the fibrous capsule (Fig. 3a) and some aggregates were also located distant from the tumor mass, within the subcutaneous tissue. In all samples from this case, all macrophages stained positive with MAH (Fig. 3b), something confirmed with lumogallion (Suppl. Fig. S1). Notably, MAH-positive cells, morphologically consistent with macrophages, were observed interspersed within the sarcoma mass (Fig. 3c-f). The nuclei of these MAH-positive cells remained unstained and exhibited a pale-yellow hue (Fig. 3e, f). In another FISS case, aggregates of macrophages containing gray-brown granular material were detected by HE staining in subcutaneous fat, distant from the main tumor mass but these findings could not be confirmed, as the aggregates were not observed in consecutive sections. In the remaining cases, positive macrophages were not detected by HE or MAH staining. Therefore, in the FISS case studied in this work the percentage of correlation MAH/lumogallion was also 100% (1/1).

In both sheep and cats, the largest crystalloid bodies were more prominently visible with MAH and/or lumogallion staining compared to HE staining (Figs. 2s-v and 3f and Suppl. Fig. S1). Smaller crystalloid bodies were typically not detected by HE but were readily identified using MAH and/or lumogallion staining. All control samples (Study cases 7–13, Suppl. Table S1) consistently tested negative for Al using both MAH and lumogallion stains (18/18; 100%).

## Discussion

This work demonstrates, for the first time, the effectiveness and simplicity of MAH staining for detecting Al in mammalian tissues. MAH uses oxidized hematoxylin (hematein) alone and provides reliable results within a short time frame, making it a valuable tool for both routine diagnostics and research. In this work, samples from Al-adjuvant-vaccinated sheep and cats with FISS were analyzed, and the results were validated using lumogallion fluorescence staining (Mold et al. 2019), further confirming MAH utility across different species.

In this study, granulomas were experimentally induced in sheep using a formulation containing inactivated BTV and AlOOH adjuvant in PBS. These granulomas were characterized by a fibrous capsule, abundant multinucleated macrophages with granular cytoplasm, central caseous necrosis, and aggregates of tertiary lymphoid follicles (Asín et al. 2019). Both MAH and lumogallion produced consistent findings, with MAH displaying a distinctive deep purple granular staining in the cytoplasm against a pale-yellow background, a hallmark feature of this technique (Havas 1986; Exley 1996). Interestingly, the nuclei remained unstained as Al particles were confined to intracytoplasmic aggregates and were absent in the nucleus (Asín et al. 2019). Similar results were observed in granulomas induced by commercial vaccines in sheep, suggesting that additional components of commercial formulations do not interfere with MAH outcomes. Both commercial and experimental vaccines showed central caseous necrosis with high Al content, as confirmed by MAH and lumogallion staining, but not with HE. Additionally, MAH detected Al in regions of severe inflammation and necrosis linked to injection site contamination following the administration of commercial Al-based vaccines. Despite the complexity of inflammatory and necrotizing components, HE could not identify Al-loaded macrophages, a limitation overcome by MAH.

The ability of MAH to detect Al in macrophages is linked to these cells capacity to accumulate Al in their cytoplasm, reaching concentrations that produce a deep purple color when MAH is applied. In tissues with trace amounts of Al, MAH may exhibit reduced sensitivity, as observed in previous studies on trout tissues (Exley 1996). In such cases, lumogallion is recommended for higher sensitivity (Mold et al. 2019). The specificity of MAH was further validated by the absence of staining in macrophages and necrotic regions of Freund’s adjuvant-induced granulomas and from granulomas associated with *Mycobacterium avium* subsp. *paratuberculosis* enteritis.

MAH effectively detected isolated macrophages in sheep regional lymph nodes, where Al tends to translocate within macrophages (Asín et al. 2019). The technique also identified Al-loaded macrophages in tertiary follicles embedded within subcutaneous granuloma, highlighting its potential for studying lymphoproliferative and/or neoplastic conditions at injection sites in both animals and humans (Roccabianca et al. 2016; Frings et al. 2021).

MAH proved particularly useful for detecting Al in FISS-affected cats, an important consideration in cases without documented vaccination histories, given that feline vaccination site guidelines have varied over recent decades (Hartmann et al. 2015).

While FISS is commonly linked to Al-adjuvanted vaccines, it can also result from other stimuli such drugs, implants, or microchips (Hartmann et al. 2015). MAH can help distinguish between vaccine-associated fibrosarcomas and spontaneous fibrosarcomas, which differ in biological behavior and prognosis (Hendrick et al., 1994). Notably, this study is the first to document MAH-positive crystalloid bodies in FISS samples, which were often overlooked in HE, but revealed by MAH. Al is known to form crystalloid bodies within post-vaccination granulomas in both, sheep and humans (Slater et al. 1982; Asín et al. 2019).

In conclusion, MAH is a simple, inexpensive, rapid, and specific histochemical technique for detecting Al in various animal tissues. This technique offers valuable insights for diagnosing Al-related conditions and should be included in reference manuals on histochemical techniques (Orchard 2013). Further research is needed to confirm whether MAH can be applied to tissues from other mammals, including humans.

## Electronic supplementary material

Below is the link to the electronic supplementary material.

Supplementary Material 1 (DOCX 721 KB)

## Data Availability

No datasets were generated or analysed during the current study.
