# Supplementary material for: Modified aluminum (Al)-hematoxylin stain for detection of Al in sheep and cat tissues: an animal model for the study of Al-associated conditions
Source: Vet Res Commun. 2025 Feb 19;49(2):108. doi: 10.1007/s11259-025-10679-y (PMC11839877; doi:10.1007/s11259-025-10679-y)
Supplement: Supplementary file 1 — Supplementary Material 1 (DOCX 721 KB) [file 11259_2025_10679_MOESM1_ESM.docx]

**Supplementary material**

**Modified aluminum (Al)-hematoxylin stain for detection of Al in sheep and cat tissues: an animal model for the study of Al-associated conditions**

Estela Pérez^1,2^, Alicia de Diego^3^, Álex Gómez^1,2^, Ana Rodríguez-Largo^1^, Marta Pérez^2,4^, Lluís Luján^1,2^*

^1^ Departamento de Patología Animal, Universidad de Zaragoza, Zaragoza, Spain.

^2^ Instituto Universitario de Investigación Mixto Agroalimentario de Aragón (IA2), Universidad de Zaragoza, 50013 Zaragoza, Spain.

^3^ Instituto Aragonés de Ciencias de la Salud (IACS), 50009 Zaragoza, Spain.

^4^ Departamento de Anatomía, Embriología y Genética, Universidad de Zaragoza, 50013 Zaragoza, Spain.

**Supplementary Table 1** Study cases with specie, number of animals, sampled tissues and stains performed

| **No.** | **Study cases** | **T/C** | **Specie** | **Animal (n)** | **Samples^a^ (n)** | **H/E (n)** | **MAH (n)** | **Lumo (n)** | **MAH/Lumo**  **(% correlation)^b^** |
| --- | --- | --- | --- | --- | --- | --- | --- | --- | --- |
| 1 | Al granuloma from experimental vaccination | T | Sheep | 6 | 10 | 10 | 10 | 5/10 | 5/5 (100%) |
| 2 | Axillar and prescapular LFNs from experimental vaccination | T | Sheep | 12 | 23 | 23 | 23 | 9/23 | 9/9 (100%) |
| 3 | Al granulomas induced by commercial vaccines | T | Sheep | 7 | 8 | 8 | 8 | 3/8 | 3/3 (100%) |
| 4 | Prescapular LFNs from animals with Al-granulomas induced by commercial vaccines | T | Sheep | 1 | 2 | 2 | 2 | 2/2 | 2/2 (100%) |
| 5 | Accidentally contaminated injection sites after using commercial Al-based vaccines | T | Sheep | 5 | 22 | 22 | 22 | 10/22 | 10/10 (100%) |
| 6 | Feline Injection Site Sarcoma (FISS) | T | Cat | 5 | 5 | 5 | 5 | 1/5 | 1/1 (100%) |
| 7 | Experimental non-Al based adjuvanted vaccine | C | Sheep | 2 | 2 | 2 | 2 | 2/2 | 2/2 (100%) |
| 8 | Axillar and prescapular LFNs from experimental non-Al based vaccine | C | Sheep | 4 | 8 | 8 | 8 | 4/8 | 4/4 (100%) |
| 9 | Experimental injection site of inactivated virus only | C | Sheep | 2 | 2 | 2 | 2 | 1/2 | 1/1 (100%) |
| 10 | Axillar and prescapular LFNs from animals injected with inactivated virus only | C | Sheep | 2 | 4 | 4 | 4 | 4/4 | 4/4 (100%) |
| 11 | Experimental injection site of PBS only | C | Sheep | 2 | 2 | 2 | 2 | 2/2 | 2/2 (100%) |
| 12 | *Mycobacterium avium* subsp. *paratuberculosis* granulomatous enteritis | C | Sheep | 3 | 3 | 3 | 3 | 2/3 | 2/2 (100%) |
| 13 | Freund adjuvant-induced granulomas | C | Rabbit | 3 | 6 | 6 | 6 | 3/6 | 3/3 (100%) |

*T*, Test case; *C*, control case; *H/E*, hematoxylin/eosin; *MAH*, modified Al-hematoxylin; *Lumo*, lumogallion; *LFNs*, lymph nodes; *PBS*, phosphate buffer saline.

^a^ Includes sections from different lesions and/or organs of the same animal (repeated sections of the same lesion have been excluded)

^b^ Percentage of correlation between MAH staining and lumogallion staining in both, positive and negative study cases.


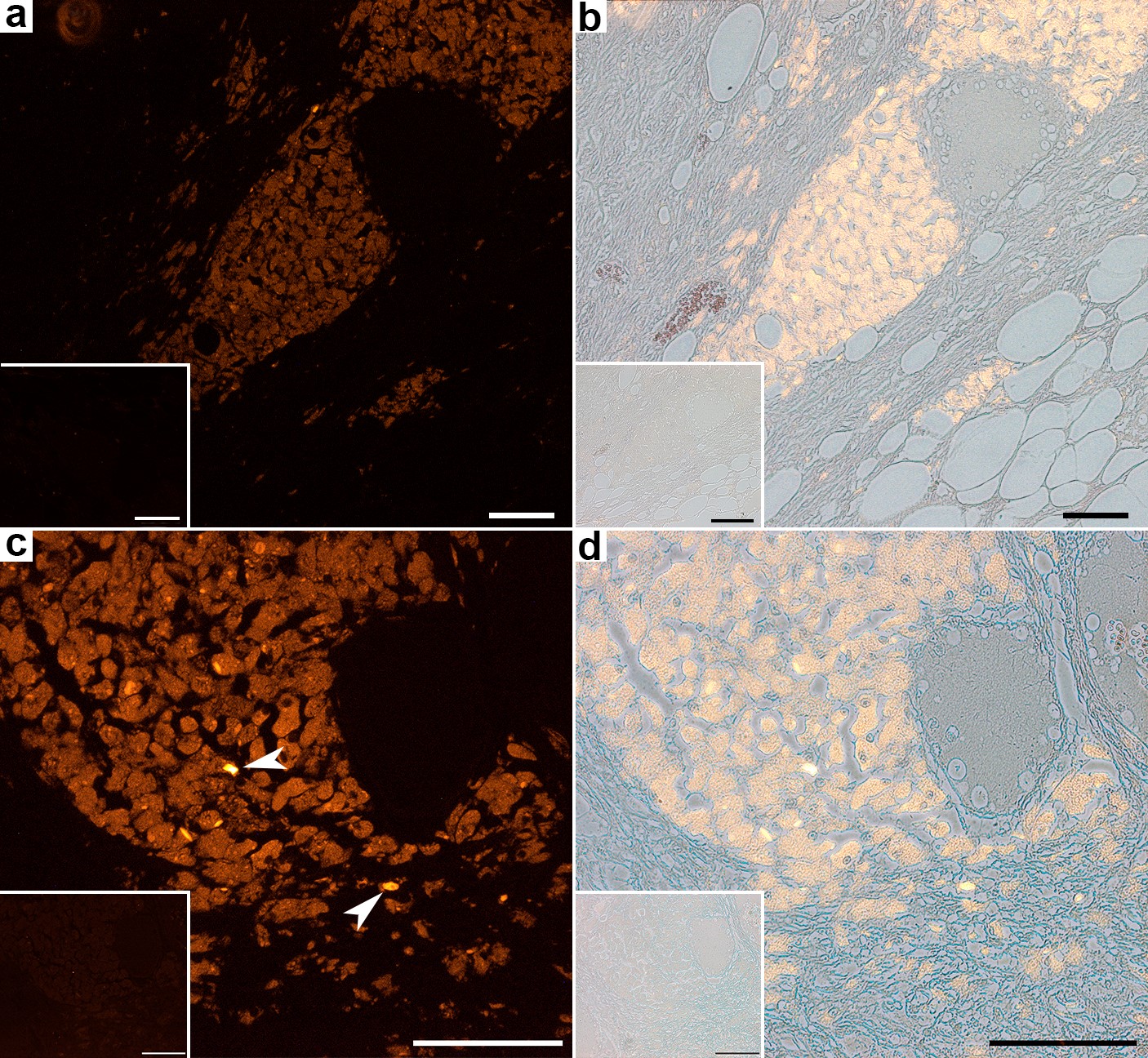


**Supplementary Fig. 1** Consecutive sections of the cat samples affected by feline injection site sarcoma (FISS) as shown in Fig. 3a. Lumogallion staining; *Insets*: autofluorescence. **a-d.** Bars 100 µm, including insets. **a.** Perivascular macrophage aggregates at the periphery of the sarcoma capsule, demonstrating positive fluorescence with a granular pattern consistent with MAH staining. **b.** Image from (a) merged with bright-field channel. **c.** Higher magnification of macrophage aggregates showing small intracytoplasmic crystalloid bodies with increased fluorescence (*arrowheads*). **d.** Image from (c) merged with bright-field channel.
